# Frequency Distribution of Mannose Binding Lectin-2 and Vitamin D Receptor Gene Variants: Putative Markers for Tuberculosis

**DOI:** 10.1155/2015/264120

**Published:** 2015-11-26

**Authors:** Anuroopa Gupta, Harish Padh

**Affiliations:** ^1^Department of Cell and Molecular Biology, B. V. Patel Pharmaceutical Education and Research Development (PERD) Centre, Sarkhej-Gandhinagar Highway, Thaltej, Ahmedabad, Gujarat 380 054, India; ^2^Institute of Science, Nirma University, Ahmedabad, Gujarat 382481, India

## Abstract

Genetic polymorphism in Mannose Binding Lectin-2 (*MBL-2*) and Vitamin D Receptor (*VDR*) is known to influence the susceptibility to tuberculosis. The objective of the present study was to evaluate the frequency distribution of the* MBL-2* promoter and structural polymorphism (−550 H/L, −221 Y/X, and +4 P/Q; R52C, G54D, and G57F) and* VDR* polymorphism (*FokI*,* BsmI*,* TaqI*, and* ApaI*) in healthy individuals of Indian population and comparative analysis with the global population. In Indian population, the frequency of* VDR* mutant alleles “f” for* FokI*, “b” for* BsmI*, “t” for* TaqI*, and “a” for* ApaI* was 25%, 54%, 30%, and 61%, respectively. The allelic frequency of* MBL-2* promoter polymorphism −550 H/L was H versus L: 32% versus 68%, −221 Y/X was Y versus X: 68% versus 32%, and +4 P/Q was P versus Q: 78% versus 22%. Mutant allelic frequencies of the* MBL-2* exon 1 D, B, and C allele were 6%, 11%, and 3%, respectively. Comparative analysis with global populations showed a noteworthy difference for* MBL-2* and* VDR* polymorphism frequency distribution, indicating the ethnic variability of Indians. The study signifies the differential distribution of susceptibility genes in Indian population, which can influence the understanding of the pathophysiology of tuberculosis in Indian population.

## 1. Introduction

*Mycobacterium tuberculosis *is one of the most ancient and life-threatening pathogens for mankind. More than one-third of the world's population harbours the tubercle bacilli asymptomatically. However, only 5–10% of the infected individuals develop the disease [1]. In 2012, one-third of the global deaths occurring due to tuberculosis were reported in India and South Africa [2]. The interindividual variation in disease susceptibility and progression is a consequence of the varied extent of host response to* M. tuberculosis. *Genetic polymorphism in Mannose Binding Lectin-2 (*MBL-2*), a central player in the innate immune response and Vitamin D Receptor (*VDR*), an immunomodulator has been found to influence the susceptibility to tuberculosis [3, 4].

The MBL-2, a pattern recognition receptor of the innate immune system [5], acts as the first line of defense against infectious agents including* M. tuberculosis*. The MBL-2 enhances the opsonization and facilitates phagocytosis of infectious agents. Variations in* MBL-2* gene influences tuberculosis susceptibility and the reports of* MBL-2* gene involvement have been contradictory [6, 7]. Low levels of MBL-2 have been associated with protection against tuberculosis [8–10] while others have reported its association with tuberculosis susceptibility [11–13]. The lower levels of MBL-2 have been attributed to the structural gene variants in the first exon of the gene:* MBL-2* D (C>T transition, Arg52Cys), B (G>A transition, Gly54Asp), and C (G>A transition, Gly57Glu) are collectively referred to as O allele while wild type is referred to as A allele [14, 15]. These results in the amino acid substitution in collagen like domain, significantly decreasing the functional MBL-2 serum levels [16]. The Single Nucleotide Polymorphisms (SNPs) in the promoter region,* MBL-2* H/L (C>G transition, −550 bp in promoter region), Y/X (G>C transition, −221 bp in promoter region), and P/Q (C>T transition, +4 bp in 5′ UTR region) [17], influence the MBL-2 transcription thus modulating the MBL-2 protein levels in serum.

The role of 1,25-dihydroxyvitamin D3, an active metabolite of vitamin D in the calcium metabolism regulation, is well established. Vitamin D3 plays a key role of an immunomodulatory hormone, whose actions are mediated through binding to a nuclear receptor, Vitamin D Receptor (VDR). Vitamin D3 activates macrophages enhancing the phagocytosis and thus restricts the* M. tuberculosis* proliferation [18]. The polymorphism in the* VDR* gene may influence the VDR activity, subsequently associating it with increased susceptibility to tuberculosis and various clinical outcomes [3, 19]. Several polymorphisms have been identified in* VDR* genes recognized by restriction endonucleases. These include* FokI* in the coding region and* BsmI*,* ApaI*, and* TaqI* in the 3′ untranslated region. The* FokI* polymorphism (F/f, C>T) in exon 2 results in* VDR* length differentiation by three amino acids producing a less active 3 amino acid-elongated* VDR* when encoded by the “f” allele [20].* BsmI *polymorphism (B/b, T>G) and* ApaI* polymorphism (A/a, T>G) occur in the intron separating exons 8 and 9.* TaqI* polymorphism (T/t, T>C) in exon 9, a synonymous SNP coding for isoleucine, enhances the VDR mRNA stability [21].

The aim of the present study was to estimate the allele and the genotype frequency distribution for* MBL-2* and* VDR* gene polymorphism in Indian population and comparative analysis of the observed data with global population reported previously to get a glimpse of ethnic variability among different populations across the world.

## 2. Materials and Methods

### 2.1. Study Population

The unrelated disease-free healthy subjects' samples were recruited at B. V. Patel PERD Centre, Ahmedabad, Gujarat. The age of healthy subjects was in the range of 19–50 years with male/female ratio of 5.4. The control individuals had no known history of tuberculosis. Healthy group comprised of individuals from western and northern parts of the country. Populations within Indian subcontinent are heterogeneous and represent elements of several ancestries. However, ancestry lineages are no longer demarcated in present geographical regions. Separate methods were used for the genotyping studies for* MBL-2* and* VDR*. Samples which did not meet particular quality parameters for analysis were not included. This resulted in variation of sample size for all the polymorphisms studied. The* MBL-2* polymorphisms were studied by sequencing of samples in 96-well plate; two of the samples failed to amplify. Genomic DNA of healthy subjects was extracted using phenol-chloroform extraction method [22]. Institutional ethical clearance and written informed consent of the blood donors were obtained prior to blood collection from the individuals.

### 2.2. Genotyping

The six SNPs in the* MBL-2* gene promoter and exon 1 (GenBank accession: rs11003125, rs7096206, rs7095891, rs5030737, rs1800450, and rs1800451) were genotyped by directly sequencing the 964 bp region amplified from genomic DNA samples of 94 disease-free healthy subjects. A preamplified PCR product of amplicon size 964 bp bracketing the region surrounding the polymorphic sites was sent for direct sequencing to Macrogen Inc., Korea, using a pair of specific primers (mentioned in Table 1).

The PCR amplification was performed for the* VDR* gene (*FokI*,* BsmI*,* TaqI,* and* ApaI*) polymorphism in a volume of 50 *µ*L in presence of 5 *µ*L of 10x PCR buffer provided with the* Taq polymerase*, 1.5 mM MgCl_2_, 0.2 mM dNTP mix, 0.2 *µ*M of each primer (mentioned in Table 1), 1-2 *µ*L of 1 U/*µ*L* Taq polymerase*, and 280 ng of genomic DNA and subjected to thermal cycler at annealing temperatures mentioned in Table 1. The amplified products were electrophoresed and visualized in ethidium bromide stained 1.5–2% agarose gels. The amplified PCR products were subjected to restriction fragment length polymorphism (RFLP) using respective restriction enzymes. The genotypes were assigned in accordance with the number of bands obtained after the digestion for each of the four polymorphisms (for details see Table 2).

### 2.3. Statistical Analysis

*MBL-2* and* VDR *alleles and genotype frequencies in disease-free healthy subjects were calculated by direct counting. The Hardy-Weinberg equilibrium (HWE) was determined. Chi-square test was performed to compare the allelic frequencies of different populations and* p* values were calculated by unconditional logistic regression and* p* < 0.05 was considered to be significant. Linkage disequilibrium and haplotype analysis was performed by Haploview version 4.2 software developed at The Broad Institute, Cambridge, MA (http://www.broadinstitute.org). The LD plot construction and haplotype frequencies were calculated using the same software [23]. The standardized disequilibrium coefficient (*D*′) analysis between the* MBL-2* SNPs was also performed using the LD plot function of this software.

## 3. Results

**Figure 1:**
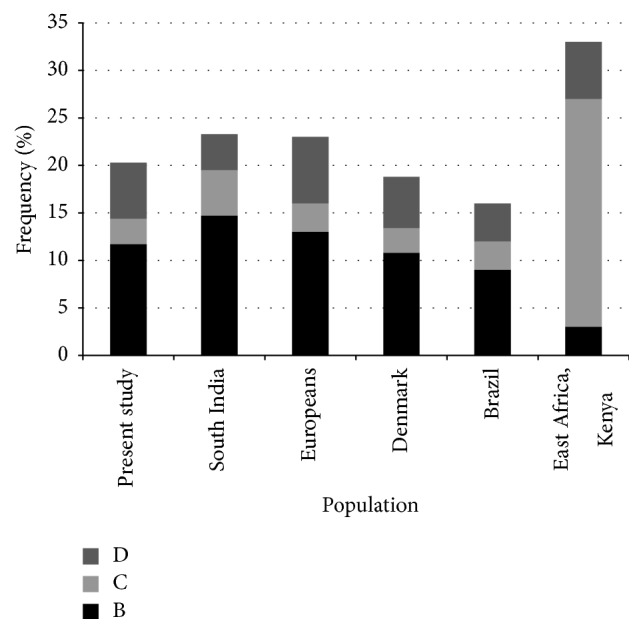
Graphical representation of* MBL-2* structural polymorphism mutant alleles (B, C, and D) variations across the global populations [4, 11, 24, 26].

In the present study,* MBL-2* promoter polymorphism (−550 H/L, −221 Y/X, and +4 P/Q) and exon 1 polymorphism (R52C: D allele, G54D: B allele, and G57F: C allele) were analyzed in 94 healthy subjects of Indian population shown in Table 3. All polymorphism with the exception of R52C were in Hardy-Weinberg equilibrium. The genotype and the allele frequency of* MBL-2* polymorphism are represented in Table 3. The allelic frequency of* MBL-2* promoter polymorphism −550 H/L was H versus L: 32% versus 68%, −221 Y/X was Y versus X: 68% versus 32%, and +4 P/Q was P versus Q: 78% versus 22%. The* MBL-2* exon 1 polymorphism mutant allelic frequency obtained for D, B, and C allele was 6%, 11%, and 3%, respectively. The observed genotype and allele frequencies in Indian population were compared with the previously reported different populations worldwide by using chi-square tests to elucidate the differences in the distribution of* MBL-2* structural variant alleles (D, B, and C) and promoter variants (Figure 1). The observations for* MBL-2* structural polymorphisms were similar to the findings reported in South Indian population [11], Europeans [24, 25], Denmark [4], and Brazilian population [26] (*p* > 0.05) (Figure 1). A significant difference was observed between the Indian (present study) and East African (Kenya) population for B (*p* = 0.002) and C (*p* < 0.0001) structural variants [24]. In comparison to* MBL-2* promoter polymorphism distribution, a statistical significant genotype frequency distribution difference between the Indians (present study) and Chinese Han population (*p* < 0.01) was observed [10].

**Figure 2:**
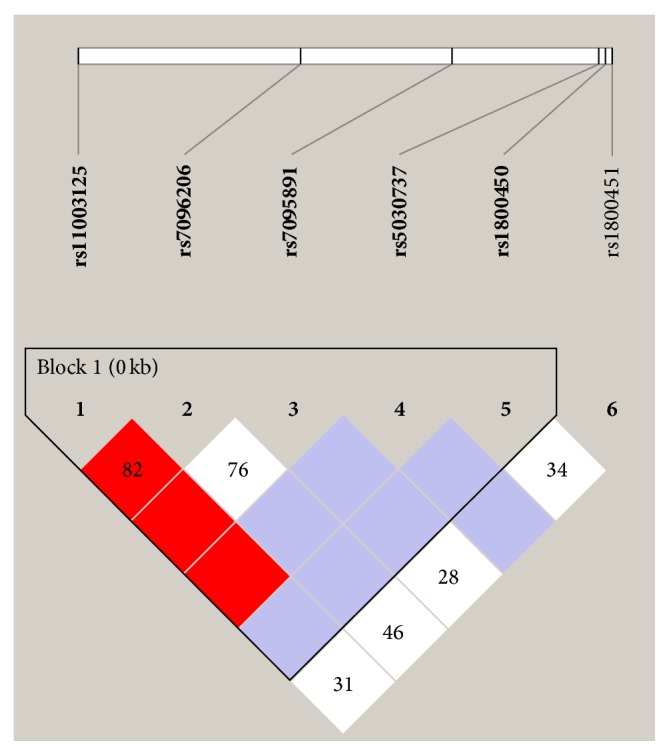
Graphical representation of Haploview LD graph of* MBL-2* gene. Squares without numbers represent D′ values of 1.0; all numbers represent the D′ value expressed as a percentile. Standard color scheme of Haploview was applied to LD color display (logarithm of odds [LOD] score ≥2 and D′ = 1, shown in bright red; LOD score ≥2 and D′ <1 shown in purple; LOD score <2 and D′ <1 shown in white).

The sequence data generated for 964 bp* MBL-2* promoter region and exon 1 was analyzed for the patterns wherein the presence of a polymorphism at one position would be consistently associated with polymorphism at one or more other positions. The examination of pairwise linkage disequilibrium (LD) between the* MBL-2* variants was performed by construction of LD plot which revealed the presence of a single haplotype block (Figure 2). Seven putative haplotypes spanning the length of the sequenced region have been identified with a frequency of more than 1% (CCCCG = 29.4%, GGCCG = 23.8%, CGTCG = 21.4%, CGCCA = 11.7%, GGCTG = 5.9%, CGCCG = 5%, and GCCCG =1.9%) [27].

**Figure 3:**
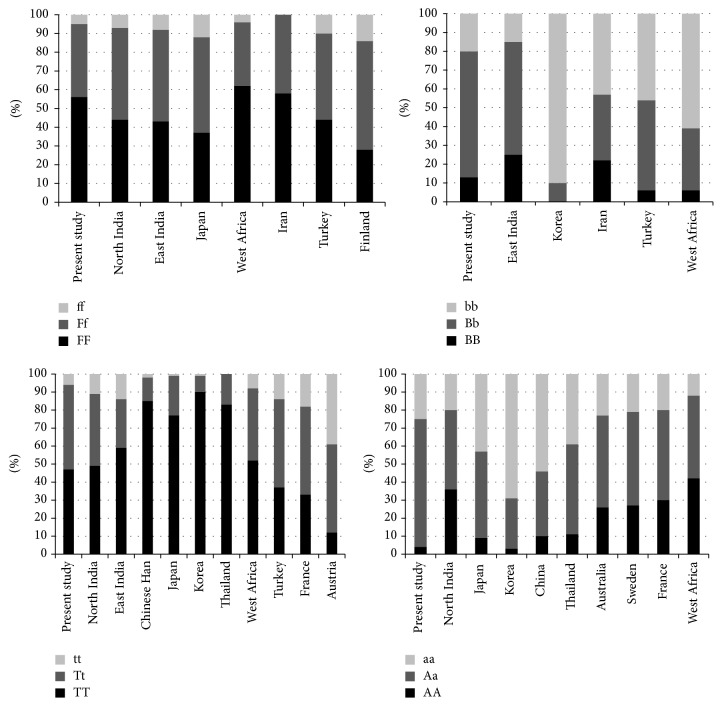
The graphical representation of* VDR* polymorphisms (*FokI, BsmI, TaqI*, and* ApaI*) variations across the global population [28, 30–33, 37, 38].

The distribution of* VDR* genotypes and allele frequencies of* FokI*,* BsmI*,* TaqI,* and* ApaI* in Indian population is shown in Table 4. The allelic frequency of “f,” “b,” “t,” and “a” alleles was 25%, 54%, 30%, and 61% obtained in Indian population. The genotype frequency of* FokI* and* TaqI* was in agreement with Hardy-Weinberg equilibrium. The observed* VDR* polymorphism genotype frequency distribution was compared individually with the different populations worldwide by using *χ* ^2^ tests (Figure 3). There was a statistically significant difference between Indians (present study) and the West Africans in* FokI*,* BsmI,* and* ApaI* polymorphism (*p* < 0.01) but nonsignificant difference in* TaqI* polymorphism (*p* = 0.142) [28]. Japanese population differs significantly from Indians (present study) in* TaqI* and* ApaI* (*p* < 0.05) and* FokI* (*p* < 0.001) polymorphism [29].* VDR* polymorphism frequency distribution differs significantly from the Korean population in* TaqI* and* BsmI* (*p* < 0.01) but is similar in* ApaI* and* FokI* (*p* > 0.05) polymorphism [30]. The frequency of the* VDR* genotypes in the present study also differs from that of studies conducted in North Indian and East Indian populations [31, 32]. Upon comparative analysis, there was a significant difference in our data from East Indians in* TaqI*,* FokI,* and* BsmI* polymorphism (*p* < 0.01) and from North Indians in* ApaI *and* FokI* except* TaqI* polymorphism (*p* < 0.01). The frequency observed was however similar to the Turkish population for* VDR FokI*,* BsmI*, and* TaqI* polymorphism [33]. There was a statistically significant difference between Indians (present study) and the Europeans (Finnish, French, Austrians, and Swedes) (*p* < 0.01), as well as with Asians (Thais and Chinese) (*p* < 0.05), for* VDR FokI*,* ApaI,* and* TaqI* polymorphism (Figure 3) [29, 31]. This demonstrates the genetic diversity within Indian population and also between different populations globally.

## 4. Discussion

The innate immunity is the first line of defense against infectious microorganisms. There are several key players of the innate immune system that interact, coordinate, and act against these infectious agents. Genetic variations in these key players can influence their mechanism of action leading to variable immune response. MBL-2 and VDR are among these key players which influence the susceptibility to* M. tuberculosis* infection and disease development. The present study involved the frequency distribution analyses of* MBL-2* and* VDR* polymorphisms, two molecules known to play a key role in tuberculosis susceptibility. The study also included haplotype study for* MBL-2* polymorphisms and evaluation of global frequency distribution disparity among different populations worldwide.

Frequency distribution of genotype and alleles of* VDR* and* MBL-2* gene varies among different ethnic population, which may lead to variable susceptibility to the infection. For* VDR FokI* “f” allele, the frequency varies from 43% in Finland to 21% in West Africa. The occurrence of “f” allele was much higher in Caucasian population. The* VDR TaqI *“t” allele frequency was found to be higher in Caucasians than Asian and African populations. The polymorphism* VDR BsmI* demonstrates a significant difference from the West African and other Asian populations.* VDR ApaI* “a” allele frequency varies from 35% in West Africa to 83% in Korea.* TaqI*,* ApaI,* and* BsmI *nonfunctional* VDR *polymorphisms show linkage disequilibrium and thus influence disease association indirectly [34]. Studies by Selvaraj et al. reported genotypes FF of* FokI*, TT of* TaqI,* and Bb of* BsmI* in males and tt of* TaqI* in females to be associated with pulmonary tuberculosis susceptibility in South Indian population [21, 35]. On the contrary, Sharma et al. (2011) reported the protective association of FF and TT genotype against the* Mycobacterium tuberculosis* infection in Central India [34]. A study conducted in Gujarati Indians living in London showed strong association of ff genotype with pulmonary tuberculosis [36]. Therefore, we are not able to draw any conclusion.

In summary, a lot of studies have been conducted through the years to understand the role of Vitamin D Receptor polymorphism in susceptibility to infectious diseases. Unfortunately, the results have been conflicting and still the role of* VDR* in tuberculosis susceptibility is not clear: the polymorphism determines susceptibility to the development of clinical disease or susceptibility to infection. Therefore the use of* VDR* polymorphism as marker for tuberculosis susceptibility is under debate.* MBL-2* variant alleles have been associated with lower serum MBL levels and contrasting associations for* MBL-2* variant alleles and mycobacterial infection have been reported in different populations.* VDR* and* MBL-2* polymorphisms' global and regional distribution, widely studied in several populations worldwide, varies significantly in each population, thus making it unique for the tuberculosis susceptibility studies. The differences in genotype and allele frequencies observed between populations could have been result of different selective pressures such as differences in diet, climate, latitude, or exposure to pathogens leading to adaptations. The efforts should be placed on a molecular understanding of the pathogenic processes, allowing a clear insight of genetic influences on the infectious diseases. The population based studies are important to reveal the population specific component to tuberculosis susceptibility which can prove effective in incorporating this into treatment and prevention strategies specifically. The study needs to be validated in larger samples to get a clearer picture of frequency distribution of* MBL-2* and* VDR* genetic variants and their association to tuberculosis susceptibility in different parts of India.

## Figures and Tables

**Table 1:** Primers and annealing temperature used in genotyping *MBL-2* and *VDR* polymorphism.

Gene	Specificity	Primers	Amplicon size	Annealing temperature
*MBL-2 Preamp*	Promoter	5′-AGAGAGGTATTTAGCACTCTGCCAG-3′ 5′-AGGACATCAGTCTCCTCATATCCC-3′	964 bp	60°C

*VDR FokI*	Exon 2	5′-AGCTGGCCCTGGCACTGACTCT-3′ 5′-GGAAACACCTTGCTTCTTCTCCCTC-3′	265 bp	60°C

*VDR BsmI*	Intron 8	5′-AAATACCTACTTTGCTGGTTTGCAGA-3′ 5′-CCCAAGGTCACAATAACTTCCTCT-3′	388 bp	60°C

*VDR ApaI*	Intron 8	5′-TGGACAGAGCATGGACAGGGAG-3′ 5′-CTCTCGGCTACTCTCGGTGATCC-3′	1391 bp	65°C

*VDR TaqI*	Exon 9	5′-TGGACAGAGCATGGACAGGGAG-3′ 5′-TTAGCTTCATGCTGCACTCAGGC-3′	485 bp	66°C

**Table 2:** The restriction digestion reaction conditions and genotype assignment after the digestion.

SNP	Incubation Temperature	Homozygous wild type	Heterozygote	Homozygous mutant	% agarose gel
*FokI*	37°C	265 bp	265 bp, 195 bp, 70 bp	195 bp, 70 bp	2%
*BsmI*	37°C	160 bp, 235 bp	388 bp, 160 bp, 235 bp	388 bp	2%
*TaqI*	65°C	485 bp	485 bp, 297 bp, 188 bp	297 bp, 188 bp	2%
*ApaI*	37°C	1174 bp, 217 bp	1391 bp, 1174 bp, 217 bp	1391 bp	1.5%

**Table 3:** The allele and genotype frequencies of *MBL-2* polymorphism among the disease-free healthy subjects in Indian population.

Polymorphism	Genotype frequency (%)			Allele frequency	
rs11003125 (H/L)	HH	HL	LL	H	L
	9 (9.6)	43 (45.7)	42 (44.7)	0.32	0.68

rs7096206 (Y/X)	YY	YX	XX	Y	X
	42 (44.7)	44 (46.8)	8 (8.7)	0.68	0.32

rs7095891 (P/Q)	PP	PQ	QQ	P	Q
	55 (58.5)	36 (38.3)	3 (3.2)	0.78	0.22

rs5030737 (A/D)	AA	AD	DD	A	D
	85 (90.4)	7 (7.4)	2 (2.2)	0.94	0.06

rs1800450 (A/B)	AA	AB	BB	A	B
	74 (78.7)	19 (20.2)	1 (1)	0.89	0.11

rs1800451 (A/C)	AA	AC	CC	A	C
	89 (94.7)	5 (5.3)	0 (0)	0.97	0.03

A/O allele	AA	AO	OO	A	O
	61 (64.9)	29 (30.9)	4 (4.2)	0.89	0.11

D, B, and C: less frequent alleles for 52-, 54-, and 57-codon polymorphism, respectively

L, X, and Q: less frequent alleles of −550, −221, and +4 polymorphism, respectively

H, Y, and P: common alleles of −550, −221, and +4 polymorphism, respectively

AA genotype represents homozygous wild genotypes for structural polymorphism

AO genotype represents heterozygous genotypes of structural polymorphism

OO genotype represents homozygous mutant genotypes of structural polymorphism as well as double heterozygous genotypes (D/B, B/C) of structural polymorphism.

**Table 4:** The genotype and allele frequencies of *VDR* polymorphism in healthy subjects in Indian population.

*VDR* polymorphism	*N*	Genotype frequency (%)			Allele frequency	
*FokI*	228	FF	Ff	ff	F	f
		128 (56.1)	88 (38.6)	12 (5.3)	0.75	0.25

*BsmI*	150	BB	Bb	bb	B	b
		19 (12.7)	100 (66.6)	31 (20.7)	0.46	0.54

*TaqI*	236	TT	Tt	tt	T	t
		110 (46.6)	110 (46.6)	16 (6.8)	0.70	0.30

*ApaI*	137	AA	Aa	aa	A	a
		5 (3.7)	98 (71.5)	34 (24.8)	0.39	0.61

(*N* = number of individuals studied.)
